# Treated incidence and baseline characteristics of substance induced psychosis in a Norwegian catchment area

**DOI:** 10.1186/1471-244X-13-319

**Published:** 2013-11-27

**Authors:** Melissa A Weibell, Inge Joa, Jørgen Bramness, Jan Olav Johannessen, Patrick D McGorry, Wenche ten Velden Hegelstad, Tor Ketil Larsen

**Affiliations:** 1 Regional Centre for Clinical Research in Psychosis, TIPS, Stavanger University Hospital, Armauer Hansensvei, 4014 Stavanger, Norway; 2 Faculty of Social Sciences, Institute of Health, University of Stavanger, Kjell Arholmsgt 41, 4021 Stavanger, Norway; 3 Norwegian Centre for Addiction Research, University of Oslo, P.O box 1039, 0315 Oslo, Norway; 4 Norwegian Institute of Public Health, P.O. box 4404, 0403 Oslo, Norway; 5 ORYGEN Research Centre, University of Melbourne, Locked bag 10, Parkville, Melbourne, VIC 3052, Australia

**Keywords:** Psychosis, Substance misuse, Substance-induced, First–episode, Incidence, Co-morbidity

## Abstract

**Background:**

Substance misuse is a well-recognized co-morbidity to psychosis and has been linked to poor prognostic outcomes in patients. Researchers have yet to investigate the difference in rates and characteristics between first-episode Substance Induced Psychosis (SIP) and primary psychosis. We aimed at comparing patients with SIP to primary psychosis patients with or without substance misuse at baseline.

**Methods:**

Thirty SIP patients, 45 primary psychosis patients with substance misuse (PS) and 66 primary psychosis patients without substance misuse (PNS) in a well-defined Norwegian catchment area were included from 2007–2011. Assessments included symptom levels (PANSS), diagnostic interviews (SCID), premorbid function scale (PAS) and global functioning (GAF f/s).

**Results:**

Treated incidence for SIP was found to be 6.5/100 000 persons per year, 9.7/100 000 persons per year for PS and 24.1/100 000 persons per year for PNS (15-65 yrs). Patients who had substance misuse (PS and SIP) were more likely to be male. Duration of Untreated Psychosis (DUP) was significantly shorter in the SIP group (5.0 wks., p = 0.003) and these had more positive symptoms on the PANSS (p = 0.049). SIP patients also did poorer on early youth academic levels on the PAS.

**Conclusions:**

Yearly treated incidence of SIP is 6.5/100 000 persons per year in a Norwegian catchment area. SIP patients have short DUPs, are more likely to be male, have more positive symptoms at baseline and poorer premorbid academic scores in early adolescence. Follow-up will evaluate stability of diagnosis and characteristics.

## Background

Distinguishing between substance-induced psychosis (SIP) and primary psychosis is crucial for understanding illness and providing optimal treatment. Substance use is widespread and causes concern for many reasons, particularly the psychotogenic properties of many substances [1-3].

Substance misuse is a well-recognized co-morbidity to schizophrenia, and rates of substance use are significantly higher in psychiatric patients than in the general population [4-7]. Studies comparing substance users to non-substance users [8-11] in psychosis have shown that persistent misuse in early course of illness is linked to higher readmission rates and more severe psychopathology [11-13]. Substance use cessation has been associated with reduced negative symptoms in first episode patients [12,14,15], highlighting the need for more research with regards to accurate diagnosis and treatment.

SIP patients are more likely to be homeless, have antisocial personality disorder, poor family support, positive family history of mental illness, more insight, more trauma as well as forensic history, and more hallucinations [16-18]. Substance misusers are more frequently male [19,20] compared to non-users in primary psychosis. Demonstrated better cognitive performance in substance abuse groups has been linked to the necessity for social skills in obtaining illegal drugs [21], and yet there is also evidence of poor academic performance [19,22].

Several substances are linked to the development of psychosis and schizophrenia [23,24] and diagnoses of substance induced psychosis have been included in ICD-10 [25] and DSM-IV [26]. The relationship between drug use and schizophrenia is probably complex [27], but meta-analysis of prospective population-based studies have estimated a doubling of risk of psychosis for cannabis, even after controlling for confounders such as reverse causation or intoxication effects [28,29]. In amphetamine users, rates of psychotic symptoms range from 5.2-100% [2,30,31], with this variation attributed to differences in sampling, methodology and extent of use.

Determining the correct diagnosis can be challenging in early-phase psychosis and is further complicated by substance misuse. Substance induced psychosis (SIP) is in DSM-IV defined as a condition in which psychotic symptoms are caused by psychoactive substances and resolve within a set time period. In order to fulfil the diagnosis, SIP must be more severe than expected from intoxication or abstinence and warrant the need for healthcare. The ICD-10 SIP diagnosis requires partial resolution of symptoms within one month and full resolution within six months, whereas the DSM-IV demands symptom remission within one month.

Diagnostic change over time is common for psychotic disorders, [3,17,32] ranging from 25-50% in SIP patients, but distinguishing and studying groups is important as they demand different treatment approaches and are often excluded from studies. Dependence or harmful use of any drug, visual hallucinations and losing contact with services have been suggested as predicting factors for SIP patients receiving a diagnosis of primary psychosis [32,33]. Literature suggests that diagnostic change is partly due to the evolution of the illness, partly our diagnostic systems where many by default get psychosis NOS diagnoses when criteria for other psychotic illnesses are not fulfilled.

Few studies have examined incidence of SIP, and these patients are often excluded from studies [24,34]. However, one study to systematically include SIP extracted data from the Danish Psychiatric Central Register, focusing on cannabis and comparing it to schizophrenia spectrum disorders [3]. The study estimated incidence of cannabis-induced psychosis in Denmark at 2.7 per 100 000 persons per year.

To our knowledge, no other study has described the treated incidence of SIP, including all substances. We wanted to investigate this in a well-defined catchment area and explore the differences in baseline characteristics compared with primary psychosis patients with and without substance misuse aiming for a better understanding of characteristics and symptom patterns. Our working hypothesis was that SIP is rare compared to other first episode psychosis (FEP) and that SIP patients have more positive symptoms than primary psychosis patients [35]. Pre-morbidly, we would expect patients with substance misuse to have better social function, but poorer functioning academically [19,22].

## Methods

The Early Identification and Treatment of Psychosis (TIPS II) study [36] is a prospective clinical trial conducted in a Norwegian catchment area, investigating first-episode psychosis (FEP) through detection teams. We work actively at recruiting patients through information campaigns and visibility at schools, health centres and doctors’ practices.

TIPS was designed to identify and follow clinically epidemiologic samples of FEP patients from Rogaland County, Norway, in the Stavanger University Hospital catchment area in the south and the Health Fonna Hospital catchment area in the north. The population of 185 337 in the south sector and 66 255 in the north sector (15–65 years) [37] live mainly in urban and suburban areas. The general medical system in Norway is nationalized, as is secondary psychiatric care. All hospital admissions and secondary care out-patient treatment are referred to the two sector hospitals.

TIPS II [36] (2002-ongoing) is a continuation of the early detection TIPS I study (1997–2000) [19,38-40]. From 2007 onwards, TIPS entered a new phase, including new patients, and for the first time also substance induced psychosis patients, dividing patients into three groups consisting of SIP patients and primary psychosis patients with and without substance misuse (PS and PNS respectively). Recruitment for this sub-study continued consecutively from August 2007 through December 2011. We map out biological, symptomatic, cognitive and demographic characteristics to assess the risk of developing schizophrenia spectrum disease in the presence of substance misuse as well as the effect of substance misuse on on-going psychotic illnesses. Follow-up is planned at 1, 2 and 5 years.

Inclusion criteria consisted of living in the catchment area; age 15–65 years; meeting DSM-IV-criteria for schizophrenia, schizophreniform psychosis, schizoaffective psychosis, delusional disorder, brief psychosis, affective disorder with mood incongruent delusions, substance induced psychosis, or psychosis not otherwise specified; being actively psychotic as measured by the Positive and Negative Syndrome Scale (PANSS) (Kay et al., 1987); not previously receiving adequate treatment for psychosis (defined as antipsychotic medication of 3.5 haloperidol equivalents for 12 weeks or until symptom remission); no neurological or endocrine disorders related to psychosis; no contraindications to antipsychotics; fluency in a Scandinavian language and IQ over 70.

The study was approved by the Regional Committee for Medical Research Ethics Health Region West and written informed consent was obtained from all study participants. The patients entered the study through the TIPS low-threshold detection team, via local general practitioners, through the hospital acute inpatient ward or outpatient clinics.

The Structured Clinical Interview for the DSM-IV (SCID) was used for diagnostic purposes in all included patients [41] and performed by a member of the research team. The team consisted of clinically experienced and trained research personnel who performed all evaluations [42]. SCID diagnoses were made on the basis of patients’ own account, co-lateral information and information from patients’ files. Strict DSM-IV criteria were applied for diagnoses. Patients were assessed by a member of the detection team within one week. If patients scored positive for abuse of drugs at intake, a longer period (> 4 weeks) of drug-free observation would be initiated before a diagnostic conclusion was made, where possible. Demographic data was collected for all study eligible patients. Diagnoses for non-consenters, that is, patients who did not sign informed consent, were either made through SCID or clinical diagnoses from patients’ files. Symptom levels at intake were measured by the PANSS [43] and global functioning by the Global Assessment of Functioning Scale (GAF) [44]. The latter scores were split into symptom (GAFs) and function scores (GAFf). Duration of untreated psychosis (DUP) was measured as the time in weeks from the first positive psychotic symptoms to the start of the first adequate treatment of psychosis (admission to the study) [45].

Pre-morbid functioning was measured by the Pre-morbid Assessment of Functioning Scale (PAS) [46], which describes four pre-morbid periods in life: Childhood (up to 11 years), Early adolescence (12–15 years), Late adolescence (16–19 years) and Adulthood (19 years and beyond). There are two pre-morbid dimensions: *social*; consisting of PAS items social isolation and peer relationships and *academic*; which contains school performance and school adaptation. Each item is scored on a Likert-type scale of 0–6, where lower numbers indicate normal, healthy functioning and higher numbers suggest pathological development.

Social functioning for the year prior to treatment was measured with the Strauss–Carpenter scale [47]. Raters were trained by rating pre-prepared case notes and audio/videotapes before entering the study assessment teams [36]. Good inter-rater reliability was achieved on major parameters in the research group previously [36], and recently; in 2012 a new score were obtained for central measures from 9 randomly selected clinical vignettes from the baseline data. Reliability of measurements for DUP was 0.8 (ICC), and for diagnostic categories; K = 0.9.

### Statistical analyses

Analyses were performed using the SPSS Statistical Program Package v 20.0 [48]. Mean values are reported with standard deviations in parentheses, with median values applied for skewed variables. DUP was log transformed prior to analysis with ANOVA. Parametrical tests were used for normally distributed data whereas non-parametric tests were applied for all univariate tests to ensure a uniform analysis strategy. Categorical variables in 2×2 crosstabs were analyzed using Fisher’s exact test. All tests are two-tailed. Outcome measures were corrected for the potential effect of gender differences applying ANCOVA (Analysis of covariance and log linear analysis, both with gender as a covariate).

Age-specific incidence rates of first-episode psychosis were calculated in yearly bands and expressed per 100 000 persons per year. The denominators for incidence calculation rates were based on estimated resident mid-year population figures for our catchment area, for each of the years 2007–2011, stratified by age [49].

## Results

**Figure 1:**
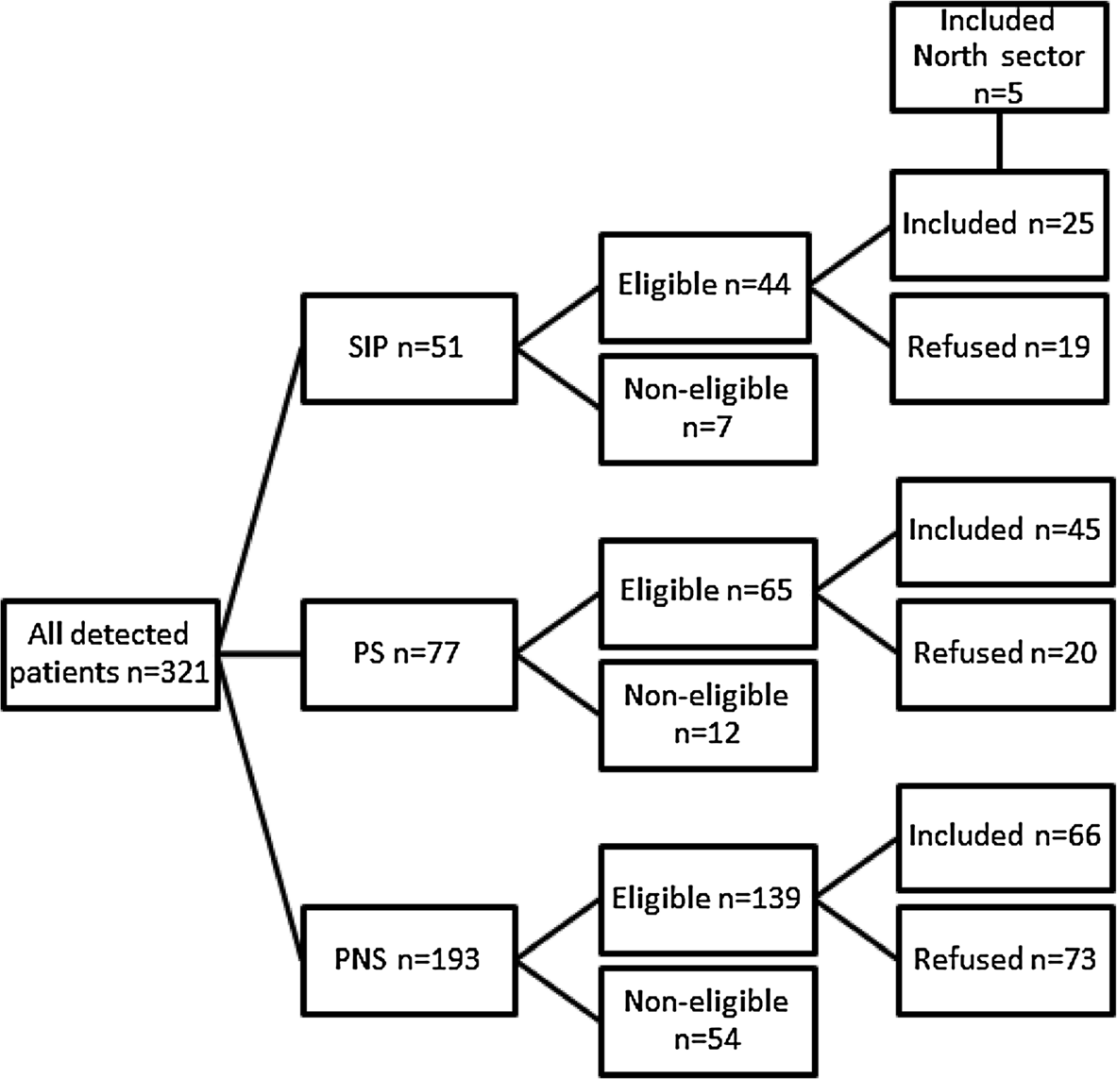
Overview of patients in the TIPS II study.

A total of 345 patients were referred to our service in from August 2007- December 2011. About half of patients were hospitalised at time of detection, and this number remained stable when analysing only included patients. 114 of these were non-eligible due to reasons such as living in a different sector, organic psychosis, previous treatment, congruent affective psychosis or non-psychotic illnesses. 51 SIP cases were identified and 25 of these were included in the TIPS II study (percentage refusing inclusion; SIP 43.2%, PS 30.1%, PNS 52.5%), along with five patients from the North Sector. Patients from the north sector did not differ from remaining patients on any parameters (Figure 1).

Treated incidence for the Stavanger University Hospital catchment area was calculated as 6.5/100 000 persons per year for SIP, 9.7/100 000 persons per year for PS and 24.1/100 000 persons per year for the PNS group (15-65 yrs) (PNS > SIP and PS; p < .005). As the rate of detection from the North sector was uncertain, five patients from this area were included to enlarge sample size, but excluded from incidence calculations.

Demographic and clinical characteristics are presented in Table 1. There were significantly more males amongst the groups of substance misusers (SIP and PS) than in the PNS group, but no significant differences with regards to age, number of children or years of education between groups. The age distribution in the included SIP sample ranged from 17 through to 53 years, with a mean age of 26.5 (SD 9.7) overall. The PS groups had a greater proportion of schizophreniform psychosis, whilst PNS patients had a higher degree of major depressive disorder. Both groups had similar rates of schizophrenia.

**Table 1:** Premorbid assessment of function (PAS) scores in patients with substance induced psychosis (SIP), psychosis without substance misuse (PNS) and psychosis with substance misuse (PS)

**PAS score**		**SIP**	**PNS**	**PS**	**p**
		**N = 30**	**N = 66**	**N = 45**	
Childhood social level	mean (SD)	1.0 (1.3)	1.3 (1.5)	1.0 (1.1)	0.436
Early adolescent social level	mean (SD)	1.4 (1.3)	1.6 (1.4)	1.2 (1.3)	0.338
Late adolescent social level	mean (SD)	1.7 (1.4)	1.5 (1.4)	1.5 (1.2)	0.831
Adult social level	mean (SD)	2.7 (1.4)	1.9 (1.6)	2.2 (1.4)	0.255
Social change adult-child	mean (SD)	1.6 (1.5)	0.7 (1.7)	1.1 (1.5)	0.212
Childhood academic level	mean (SD)	2.2 (1.2)	1.6 (1.4)	2.2 (1.7)	0.274
Early adolescent academic level	mean (SD)	3.1 (1.3)	2.1 (1.4)	2.8 (1.7)	0.027*
Late adolescent academic level	mean (SD)	3.0 (1.4)	2.2 (1.4)	2.8 (1.6)	0.058
Academic change	mean (SD)	0.8 (1.4)	0.7 (1.7)	0.8 (1.6)	0.917

* p < 0.05.

SIP patients scored higher on the PANSS positive sum score (p = 0.049). GAF scores did not reveal any differences. DUP varied greatly between groups with SIP patients having a median DUP of 5.0 weeks compared to PNS patients (25.5 weeks) and PS patients (20.0 weeks, p = 0.003). DUP remained significantly shorter in SIP patients, even when including patients who did not consent to inclusion (Table 2).

**Table 2:** Baseline characteristics of patients with substance induced psychosis (SIP), psychosis without substance misuse (PNS) and psychosis with substance misuse (PS) including Positive and negative symptom score (PANSS)

	**SIP**	**PNS**	**p**^**a**^	**PS**	**p**^**b**^	**p**^**c**^
	**n = 30**	**n = 66**		**n = 45**		
	**n (%)**	**n (%)**		**n (%)**		
Male gender	21 (70.0%)	32 (48.5%)	0.049*	32 (71.1%)	0.918	
Single marital status	24 (80.0%)	36 (63.2%)	0.142	36 (80.0%)	1.000	
Form of living (flat)	21 (70.0%)	53 (80.3%)	0.266	34 (75.6%)	0.594	
Married/co-habiting	3 (10.0%)	17 (25.8%)	0.078	6 (13.3%)	0.663	
	**Mean (SD)**	**Mean (SD)**		**Mean (SD)**		
Age (all detected)	26.6 (8.8)	27.4 (12.3)		25.2 (8.2)		0.281
Included sample	25.2 (8.3)	27.3 (11.0)		26.2 (8.4)		0.382
PANSS positive subscale sum	18.3 (4.7)	15.4 (5.8)		18.0 (5.6)		0.049*
PANSS negative subscale sum	12.7 (4.7)	13.1 (5.4)		13.9 (6.2)		0.582
PANSS general subscale sum	30.8 (5.0)	30.7 (8.6)		31.3 (9.1)		0.9344
PANSS total sum	61.9 (10.7)	59.4 (15.3)		63.5 (17.1)		0.713
GAF symptoms scores	33.0 (7.0)	33.7 (8.8)		31.7 (8.1)		0.579
GAF function scores	39.1 (10.1)	42.7 (12.9)		37.8 (10.8)		0.257
	**Median (range)**	**Median (range)**		**Median (range)**		
Duration of untreated psychosis (weeks)	5.0 (0–416)	25.5 (0–1092)	0.005**	20.0 (0–1508)	0.028*	0.003**

*p < 0.05.

**p < 0.01.

^a^between SIP and PNS (categorical variables).

^b^between SIP and PS (categorical variables).

^c^across all groups (continuous variables).

On the PAS, SIP patients did worse than PNS patients with regards to early adolescent academic levels (p = 0.027) and tended towards poorer performance on late adolescent academic levels (p = 0.058) (Table 1).

With respect to diagnostic distribution, Psychosis NOS represented a large part of PS patients (46.7%) whereas PNS diagnoses were more evenly distributed (Table 3).

**Table 3:** Diagnostic distribution of included sample of patients with substance induced psychosis (SIP), psychosis without substance misuse (PNS) and psychosis with substance misuse (PS)

**DSM-IV diagnosis**			**SIP**	**PNS**	**PS**
			**N = 30**	**N = 66**	**N = 45**
Alcohol-induced psychotic disorder	291.50	N (%)	1 (3.3%)		
Alcohol-induced mood disorder	291.80	N (%)	1 (3.3%)		
Substance-induced psychotic disorder, with delusions	292.11	N (%)	14 (46.7%)		
Substance-induced psychotic disorder, with hallucinations	292.12	N (%)	14 (46.7%)		
Schizophrenia, paranoid type	295.30	N (%)		13 (19.7%)	12 (26.6%)
Schizophreniform disorder	295.40	N (%)		3 (4.5%)	11 (24.2%)
Schizoaffective disorder	295.70	N (%)		8 (12.1%)	2 (4.4%)
Major depressive disorder	296.22, 296.24, 296.34	N (%)		14 (21.2%)	2 (4.4%)
Bipolar disorder	296.40, 296.89	N (%)		4 (6.0%)	2 (4.4%)
Delusional disorder	297.10	N (%)		7 (10.6%)	3 (8.6%)
Brief psychotic disorder	298.80	N (%)		2 (3.0%)	2 (4.4%)
Psychotic disorder NOS	298.90	N (%)		13 (19.7%)	(46.7%)

At inclusion SIP patients had less suicide thoughts, plans or attempts (p = 0.039). There were no differences between groups with regards to PTSD or significant life events (p > .05) The Strauss-Carpenter scale revealed that the PS group received more social support compared to the PNS group (p = 0.010). Furthermore, significantly more SIP patients had admissions lasting less than six months compared to the PNS group (p = 0.042).

Most of the patients in our sample used cannabis or amphetamines with SIP patients using significantly more opiates (Table 4).

**Table 4:** Distribution of substance use in patients with substance induced psychosis (SIP) and psychosis with substance use (PS)

		**PNS**	**PS**	**SIP**	**p**
Alcohol	N (%)	46 (71.2%)	41 (93.3%)	29 (96.7%)	0.004^a^*, 0.004^b^*
Cannabis	N (%)		39 (86.7%)	20 (80.0%)	p = 0.506
Stimulants	N (%)		33 (73.3%)	20 (80.8%)	p = 0.533
Cocaine	N (%)		11 (24.4%)	8 (32.0%)	p = 0.496
Opiates	N (%)		9 (20.0%)	11 (44%)	p = 0.033*
Sedatives	N (%)		14 (31.3%)	8 (32.0%)	p = 0.939
Other substances	N (%)		15 (33.3%)	11 (44%)	p = 0.376

*p < 0.05.

^a^between PNS and PS (categorical variables).

^b^between PNS and SIP (categorical variables).

## Discussion

Our study showed that the treated incidence of SIP and PS was lower than in the PNS group. There were no differences in age, marital status, living arrangements or GAF scores between groups, but significantly more SIP patients were male and tended to have less lasting relationships compared to PNS patients. SIP patients had significantly shorter DUPs than the primary psychosis groups; 5 vs. 20 and 26 weeks; and more positive psychosis symptoms. Pre-morbidly, SIP patients had the poorest scores on early adolescent academic levels, suggesting that there are problems in this group from adolescence on.

Most of the patients in our sample reported use of cannabis, amphetamines, or a combination of several substances. This is consistent with amphetamines and cannabis being the two most prevalent illegal substances used in Norway, with around 15% of people (16–64 years) having tried cannabis and 1-2% having tried amphetamines [48]. SIP patients however, appeared to have a higher frequency of opiate use than the PS group, possibly indicating heavier substance use.

The TIPS project involves extensive detection work in a well-defined catchment area where all patients are admitted to our hospital. The detection team is based in the acute admissions ward at the hospital and liaises with general practitioners, schools and youth centres. Numerous information campaigns have significantly increased awareness of psychosis in our catchment area [36]. This is further strengthened by a stable population with low migration numbers, as one gets a non-selected/”real world scenario”. Furthermore, the mix between rural and urban environments is viewed as very representative of the general Norwegian population [49]. Still, we consider this a study of treated incidence rather than true incidence of first episode psychosis (FEP) as our calculations are based on help seeking behaviour. With regards to primary FEP it is reasonable to assume that most patients will require clinical attention. We do not know whether this is the case with SIP. Our estimate of treated incidence of 6.5/100 000 persons per year of SIP is probably a conservative estimate and requires replication.

Diagnosing psychotic disorders using DSM-IV criteria may be challenging in a clinical setting when psychosis co-occurs with substance misuse or with an inadequate substance-free period. In such cases, the default DSM-IV diagnosis would be psychosis NOS, due to the lack of fulfilment of criteria for serious mental illness [44]. This is a limitation of our study, due to unwillingness to over-diagnosing primary psychotic disorders in patients possibly intoxicated or in withdrawal. This is also seen in other studies [7,33] and may partially explain the high prevalence of psychosis NOS.

Literature shows that a substantial part of SIP patients will at some stage be diagnosed with a primary psychotic disorder, [3,17,32] but with our current diagnostic systems, we might limit our ability to accurately detect and separate SIP from primary psychosis illnesses. This further emphasizes the need for samples such as ours where one can follow patients prospectively with the aim of finding characteristics that will allow us to separate the two groups and determine whether the diagnostic drift is in fact a drift or merely a case of inaccurate diagnosis. This article presents baseline data and diagnostic stability will be addressed in the longitudinal follow-up of our cohort.

Our refusal rate was high in all groups, reflecting the arduousness of recruiting this patient-group into research as well as into the health care-system, as shown in other studies involving this group of patients [50,51]. In our sample, around half of SIP patients (53.5%) were hospitalized at time of inclusion, thus making it easier for the research team to observe patients substance-free. The study is strengthened by having demographic data on all eligible patients. There is some uncertainty in the diagnoses of non-consenters as these may be based on initial interviews where status of substance use is unknown or clinical diagnoses are based on discharge letters.

Many patients are detected, assessed, but then discharge themselves against medical advice, or after the psychosis has resolved, without leaving any means of contact, possibly due to lack of insight or being too influenced by symptoms such as paranoia to be able to accept offers of treatment. This has also been found by others [52], and is confirmed in our sample where SIP patients have shorter admissions than the other groups. However, it should be kept in mind that even short admissions provide opportunities for identifying patients at risk and planning tailored interventions.

Hallucinations following the intake of drugs are quite common. Different assessment methods make it difficult to compare study rates. One study applying the Brief Psychiatric Rating Scale [2], found that 23% had experienced a symptoms of suspiciousness, unusual thought content or hallucinations in the past year. DSM-IV criteria only consider hallucinations due substance intake as psychotic when they occur in the absence of insight, in contrast to primary psychosis disorders. Most likely, this is to avoid over-diagnosing psychosis following drugs that commonly cause hallucinations. However, several studies suggest that hallucinations are a key feature of SIP, with SIP patients being more likely to consider their symptoms psychotic [16,17]. Intact insight in psychosis has also been reported as a common feature among most methamphetamine users with psychotic symptoms [53]. By excluding hallucinations in patients with insight for diagnostic purposes, DSM-IV risks excluding patients with distressing symptoms from receiving proper psychiatric treatment as their symptoms may be ruled clinically insignificant.

## Conclusions

It is possible that even our comprehensive detection system in a limited catchment area could overlook some SIP patients due to difficulties in detection and engaging them in our services. This is further complicated by obstacles in diagnosing and assessing patients. Applying different test-instruments, diagnostic standards and cut-off criteria make it difficult to draw definitive conclusions with regards to cause, effect and risk and thus do not provide us with the solid platform essential to enhance our knowledge of these patients.

Our results suggest that that SIP is associated with more acute onset due to a proximal trigger (substance misuse) and that more severe symptoms propel patients into care more rapidly. SIP patients performed poorer academically than the two other groups in early adolescence, a finding that is supported by other studies [19,22] and would, in itself potentially increase risk of substance use. Our interpretation is, however, limited by not having data on onset of substance misuse.

Guidelines for assessment and treatments of these patients as a group are needed as SIP patients may need different medication, brief medication or no medication and they may be more susceptible to the adverse effects of antipsychotic treatment. Future research should focus on a common methodology to determine incidence rates and characteristics of SIP patients necessary to inform best delivery of care, but also to shed light on the trajectory from substance misuse to primary psychosis.

## Competing interests

None of the authors declare any competing interests.

This work was supported by the National Research council and Stavanger University Hospital.

The funding source had no role in the study design, in collection, analyses and interpretation of data, in writing the report, or in the decision to submit the paper for publication.

## Authors’ contributions

TKL, IJ and JOJ originally designed the study and wrote the protocol. MW managed the literature searches and analyses as well as statistical analysis and writing of the manuscript. All authors contributed to and have approved the final manuscript.

## Pre-publication history

The pre-publication history for this paper can be accessed here:

http://www.biomedcentral.com/1471-244X/13/319/prepub

## References

[B1] Satel SL Edell WS Cocaine-induced paranoia and psychosis proneness Am J Psychiatry 1991 148 12 1708 1711 1957934 10.1176/ajp.148.12.1708

[B2] McKetin R McLaren J Lubman DI Hides L The prevalence of psychotic symptoms among metamphetamine users Addiction 2006 101 1473 1478 10.1111/j.1360-0443.2006.01496.x 16968349

[B3] Arendt M Rosenberg R Foldager L Perto G Munk-jorgensen P Cannabis-induced psychosis and subsequent schizophrenia-spectrum disorders: follow-up study of 535 incident cases Br J Psychiatry 2005 187 510 515 10.1192/bjp.187.6.510 16319402

[B4] Regier DA Farmer ME Rae DS Locke BZ Keith SJ Judd LL Goodwin FK Comorbidity of mental disorders with alcohol and other drug abuse- results from the epidemiologic catchment area (EDC) study J Am Med Assoc 1990 264 19 2511 2518 10.1001/jama.1990.03450190043026 2232018

[B5] Jablensky A McGrath J Herrman H Castle D Gureje O Evans M Carr V Morgan V Korten A Harvey C Psychotic disorders in urban areas: an overview of the study on low prevalence disorders Aust N Z J Psychiatry 2000 34 2 221 236 10.1080/j.1440-1614.2000.00728.x 10789527

[B6] Cantwell R Brewin J Glazebrook C Dalkin T Fox R Medley I Harrison G Prevalence of substance misuse in first-episode psychosis Br J Psychiatry 1999 174 150 153 10.1192/bjp.174.2.150 10211169

[B7] Barnett JH Werners U Secher SM Hill K Brazil R Masson K Pernet DE Kirkbride JB Murray GK Bullmore ET Substance use in a population-based clinic sample of people with first-episode psychosis Br J Psychiatry 2007 190 515 520 10.1192/bjp.bp.106.024448 17541112

[B8] Hambrecht M Häfner H Sustance abuse and the onset of schizophrenia Biol Psychiatry 1996 40 1155 1163 10.1016/S0006-3223(95)00609-5 8931919

[B9] Sorbara F Liraud F Assens F Abalan F Verdoux H Substance use and the course of early psychosis: a 2-year follow up of first-admitted subjects Eur Psychiatry 2003 18 133 136 10.1016/S0924-9338(03)00027-0 12763300

[B10] Wade D Harrigan S Edwards J Burgess PM Whelan G McGorry PD Substance misuse in first-episode psychosis: 15-month prospective follow-up study Br J Psychiatry 2006 189 229 234 10.1192/bjp.bp.105.017236 16946357

[B11] Wade D Harrigan S McGorry PD Burgess PM Whelan G Impact of severity of substance use disorder on symptomatic and funcitonal outcome in young individuals with first-episode psychosis J Clin Psychiatry 2007 68 5 767 774 10.4088/JCP.v68n0517 17503988

[B12] Turkington A Mulholland CC Rushe TM Anderson R McCaul R Barrett SL Barr RS Cooper SJ Impact of persistent substance misuse on 1-year outcome in first-episode psychosis Br J Psychiatry 2009 195 242 248 10.1192/bjp.bp.108.057471 19721115

[B13] Addington J Addington D Patterns, prdictors and impact of substance use in early psychosis: a longitudinal study Acta Psychiatr Scand 2007 115 304 309 10.1111/j.1600-0447.2006.00900.x 17355521

[B14] González-Pinto A Alberich S Barbeito S Gutierrez M Vega P Ibáñez B Haidar MK Vieta E Arango C Cannabis and first-episode psychosis: different long-term outcomes depending on continued or discontinued use Schizophr Bull 2011 37 3 631 639 10.1093/schbul/sbp126 19915168 PMC3080669

[B15] Lambert M Conus P Lubman DI Wade D Yuen H Moritz S Naber D McGorry PD Schimmelmann BG The impact of substance use disorders on clinical outcome in 643 patients with first-episode psychosis Acta Psychiatr Scand 2005 112 2 141 148 10.1111/j.1600-0447.2005.00554.x 15992396

[B16] Serper MR Chou JC-Y Allen MH Czobor P Cancro R Symptomatic overlap of cocaine intoxication and acute schizophrenia at emergency presentation Schizophr Bull 1999 25 2 387 394 10.1093/oxfordjournals.schbul.a033386 10416739

[B17] Caton CLM Drake RE Hasin DS Dominguez B Shrout PE Samet S Schanzer B Differences between early-phase primary psychotic disorders with concurrent substance use and substance-induced psychosis Arch Gen Psychiatry 2005 62 137 145 10.1001/archpsyc.62.2.137 15699290

[B18] Fraser S Hides L Philips L Proctor D Lubman DI Differentiating first episode substance induced and primary psychotic disorders with concurrent substance use in young people Schizophr Res 2012 136 1–3 110 115 22321667 10.1016/j.schres.2012.01.022

[B19] Larsen TK Melle I Auestad B Friis S Haahr U Johannessen JO Opjordsmoen S Rund BR Simonsen E Vaglum P Substance abuse in first-episode non-affective psychosis Schizophr Res 2006 88 55 62 10.1016/j.schres.2006.07.018 16971092

[B20] Sevy S Robinson DG Napolitano B Patel RC Gunduz-Bruce H Miller R McCormack J Lorell BS Kane J Are cannabis use disorders associated with an earlier age at onset of psychosis? A study in first episode schizophrenia Schizophr Res 2012 120 101 107 20471224 10.1016/j.schres.2010.03.037 PMC2900481

[B21] Løberg E-M Hugdahl K Cannabis use and cognition in schizophrenia Front Human Neurosci 2009 3 1 8 10.3389/neuro.09.053.2009 PMC2786315 19956405

[B22] Ringen PA Melle I Birkenaes AB Engh JA Faerden A Vaskinn A Friis S Opjordsmoen S Andreassen OA The level of illicit drug use is related to symptoms and premorbid functioning in severe mental illness Acta Psychiatr Scand 2008 118 297 304 10.1111/j.1600-0447.2008.01244.x 18759810

[B23] Zammit S Allebeck P Andreasson S Lundberg I Lewis G Self reported cannabis use as a risk factor for schizophrenia in Swedis conscripts of 1969: historical cohort study Br Med J 2002 325 1199 10.1136/bmj.325.7374.1199 12446534 PMC135490

[B24] Arseneault L Cannon M Poulton R Murrey R Caspi A Moffit TE Cannabis use in adolescence and risk for adult psychosis: longitudinal prospective study Br Med J 2002 325 1212 1213 10.1136/bmj.325.7374.1212 12446537 PMC135493

[B25] WHO The ICD-10 Classification of Mental and Behavioural Disorders 1992 Geneva: World Health Organisation

[B26] American Psychiatric Association Diagnostic and Statistical Manual of Mental Disorders 2000 4 Washington DC: American Psychiatric Association

[B27] Bramness JG Gundersen OH Guterstam J Rognli EB Konstenius M Løberg E-M Medhus S Tanum L Franck J Amphetamine-induced psychosis - a separate diagnostic entity or primary psychosis triggered in the vulnerable BMC Psychiatry 2012 12 doi: 10.1186/1471-244X-12-221 10.1186/1471-244X-12-221 PMC3554477 23216941

[B28] Arseneault L Cannon M Witton J Murrey RM Causal association between cannabis and psychosis: examination of the evidence Br J Psychiatry 2004 184 110 117 10.1192/bjp.184.2.110 14754822

[B29] Moore THM Zammit S Lingford-Hughes A Barnes TRE Jones PB Burke M Lewis G Cannabis use and risk of psychotic or affective mental health outcomes: a systematic review Lancet 2007 370 319 238 10.1016/S0140-6736(07)61162-3 17662880

[B30] Smith MJ Thirtalli J Abdallah AB Murray RM Cottler LB Prevalence of psychotic symptoms in substance users: a comparison across substances Compr Psychiatry 2009 50 3 245 250 10.1016/j.comppsych.2008.07.009 19374969 PMC2743957

[B31] Glasner-Edwards S Mooney LJ Marinelli-Casey P Hillhouse M Ang A Rawson R Clinical course and outomes of metamphetamine-dependent adults with psychosis J Subst Abus Treat 2008 35 4 445 450 10.1016/j.jsat.2007.12.004 18294802

[B32] Crebbin K Mitford E Paxton R Turkington D First-episode drug-induced psychosis: a medium term follow up study reveals a high risk group Soc Psychiatry Psychiatr Epidemiol 2009 44 710 715 10.1007/s00127-008-0490-2 19183816

[B33] Caton CLM Hasin DS Shrout PE Drake RE Dominguez B First MB Samet S Schnauzer B Stability of early-phase primary psychotic disorders with concurrent substance use and substance-induced psychosis Br J Psychiatry 2007 190 105 111 10.1192/bjp.bp.105.015784 17267925

[B34] Henquet C Krabbendam L Spauwen J Kaplan C Lieb R Wittchen H-U Os J Prospective cohort study of cannabis use, predisposition for psychosis and psychotic symptoms in young people Br Med J 2005 330 11 14 10.1136/bmj.38267.664086.63 15574485 PMC539839

[B35] Harris D Batki S Stimulant psychosis: symptom profile and acute clinical course Am J Addict 2000 9 1 28 37 10.1080/10550490050172209 10914291

[B36] Joa I Johannessen JO Auestad B Friis S McGlashan T Melle I Opjordsmoen S Simonsen E Vaglum P Larsen TK The key to reducing duration of untreated first psychosis: information campaigns Schizophr Bull 2008 34 3 466 472 17905788 10.1093/schbul/sbm095 PMC2632428

[B37] Statistics Norway Population Data 2011 http://statbank.ssb.no/statistikkbanken/Default_FR.asp?PXSid=0&nvl=true&PLanguage=0&tilside=selecttable/MenuSelS.asp&SubjectCode=02

[B38] Melle I Larsen TK Haahr U Friis S Johannessen JO Opjordsmoen S Simonsen E Rund BR Vaglum P McGlashan TH Reducing the duration of untreated psychosis in first episode psychosis Arch Gen Psychiatry 2004 61 2 143 150 10.1001/archpsyc.61.2.143 14757590

[B39] Larsen TK Johannessen JO McGlashan TH Friis S Guldberg C Haahr U Horneland M Melle I Opjordsmoen S Simonsen E Reducing duration fo untreated first psychosis: changes in patient characteristics at presentation Am J Psychiatr 2001 158 11 1917 1919 10.1176/appi.ajp.158.11.1917 11691702

[B40] Johannessen JO Larsen TK Joa I Melle I Friis S Opjordsmoen S Lund BR Simonsen E Vaglum P McGlashan T Pathways to care for first-episode psychosis in an early detection healthcare sector. Part of the Scandinavian TIPS study Br J Psychiatry 2005 187 24 28 10.1192/bjp.187.48.s24 16055803

[B41] First MB Spitzer RL Gibbon M Williams JBW Structured Clincial Interview for DSM-IV Axis I Disorders-Patient Edition (SCID I/P, Version 2.0) 1995 New York: New York State Pscyhiatric Institute, Biometrics Research Department

[B42] Friis S Larsen TK Melle I Al E Methodological pitfalls in early detection studies - the NAPE lecture 2002 Acta Psychiatr Scand 2003 107 3 9 10.1034/j.1600-0447.2003.02600.x 12558535

[B43] Kay SR Fiszbein A Opler LA The positive and negative syndrome scale (PANSS) for schizophrenia Schizophr Bull 1987 13 261 276 10.1093/schbul/13.2.261 3616518

[B44] American Psychiatric Association Diagnostic and Statistical Manual of Mental Disorders (Dsm-Iv) 1994 4 Washington DC: American Psychiatric Association

[B45] Larsen TK McGlashan TH Moe LC First-episode schizophrenia: I Early course parameters Schizophrenia Bull 1996 22 2 241 256 10.1093/schbul/22.2.241 8782284

[B46] Cannon-Spoor HE Potkin SG Wyatt RJ Measurement of premorbid adjustment in chronic schizophrenia Schizophr Bull 1982 8 470 484 10.1093/schbul/8.3.470 7134891

[B47] Strauss JS Carpenter WT The prediction of outcome in schizophrenia II Relationships between predictor and outcome variables: a report from the WHO pilot study of schizophrenia Arch Gen Psychiatry 1974 31 37 42 10.1001/archpsyc.1974.01760130021003 4835985

[B48] EMCDDA 2010 Annual Report on the State of the Drugs Problem in Europe 2010 Lisbon: European monitoring centre for drugs and drug addiction

[B49] Johannessen J McGlashan T Larsen T Horneland M Joa I Mardal S Kvebæk R Friis S Melle I Opjordsmoen S Early detection strategies for untreated first-episode psychosis Schizophr Res 2001 51 39 46 10.1016/S0920-9964(01)00237-7 11479064

[B50] Tarrier N Khan S Cater J Picken A The subjective consequences of suffering a first episode psychosis: trauma and suicide behaviour Soc Psychiatry Psychiatr Epidemiol 2007 42 29 35 10.1007/s00127-006-0127-2 17082897

[B51] Kavanagh DJ Young R White A Saunders JB Wallis J Shockley N Jenner L Clair A A brief motivational intervention for substance misuse in recent-onset psychosis Drug Alcohol Rev 2004 23 151 155 10.1080/09595230410001704127 15370020

[B52] Mordal J Holm B Gossop M Romøren M Mørland J Bramness JG Psychoactive substance use among patients admitted to an acute psychiatric ward: laboratory findings and associations with clinical characteristics Nord J Psychiatry 2011 65 208 215 10.3109/08039488.2010.527014 21047195

[B53] Matsumoto T Kamijo A Miyakawa Y Endo K Yabana T Kishimoto H Okudaira K Iseki E Sakai T Kosaka K Metamphetamine in Japan: the consequences of metamphetamine abuse as a funciton of route of administration Addiction 2002 97 809 817 10.1046/j.1360-0443.2002.00143.x 12133119

